# Supplementary material for: Structure insights into selective coupling of G protein subtypes by a class B G protein-coupled receptor
Source: Nat Commun. 2022 Nov 5;13:6670. doi: 10.1038/s41467-022-33851-3 (PMC9637140; doi:10.1038/s41467-022-33851-3)
Supplement: Supplementary file 1 — Supplementary Information [file 41467_2022_33851_MOESM1_ESM.pdf]

Supplementary Information

Structure insights into selective coupling of G protein subtypes by a class B G protein-coupled receptor

Brief description of what this file includes:

Supplementary Fig. 1 Purification and characterization of the UCN1-CRF2R-G_{11} and UCN1-CRF2R-G_o complexes.

Supplementary Fig. 2. Flowchart of cryo-EM data analysis of the UCN1-CRF2R-G_{11} complex.

Supplementary Fig. 3. Flowchart of cryo-EM data analysis of the UCN1-CRF2R-G_o complex.

Supplementary Fig. 4. Cryo-EM density maps of the UCN1-CRF2R–G protein structures.

Supplementary Fig. 5. G protein activation and signaling assays of wild-type (WT) and mutant CRF2Rs and the schematic figure of important residues on CRF2R and G protein for formation of the G protein subtype-specific interactions.

Supplementary Fig. 6. G protein activation and signaling assays of wild-type (WT) and mutant CRF2Rs and expression levels of CRF2R mutations in HEK293 cells.

Supplementary Table 1. Cryo-EM data collection, refinement and validation statistics.

Supplementary Table 2. Effects of mutations on UCN1-induced activation of wild-type and mutant CRF2R.

Supplementary Table 3. List of primer sequences for site-direct mutagenesis studies.

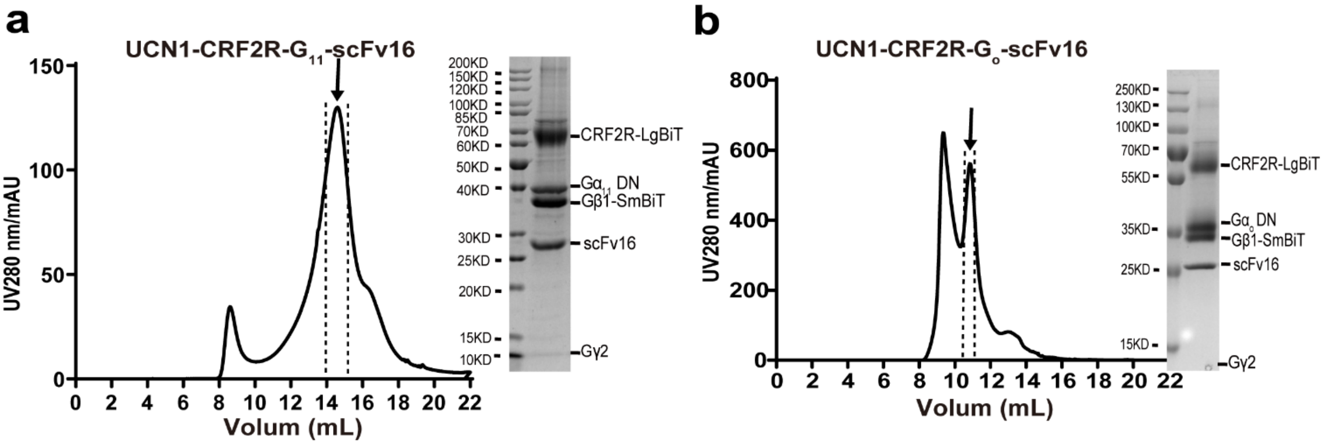

Supplementary Fig. 1. Purification and characterization of the UCN1-CRF2R-G_{11} and UCN1-CRF2R-G_o complexes. **(a)** Size-exclusion chromatography elution profiles of the purified UCN1-CRF2R-G_{11} complex on Superose6 Increase 10/300GL (left panel) and SDS-PAGE analysis of UCN1-CRF2R-G_{11} complex (right panel). **(b)** Size-exclusion chromatography elution profiles of the purified UCN1-CRF2R-G_o complex on Superdex200 Increase 10/300GL (left panel) and SDS-PAGE analysis of UCN1-CRF2R-G_o complex (right panel).

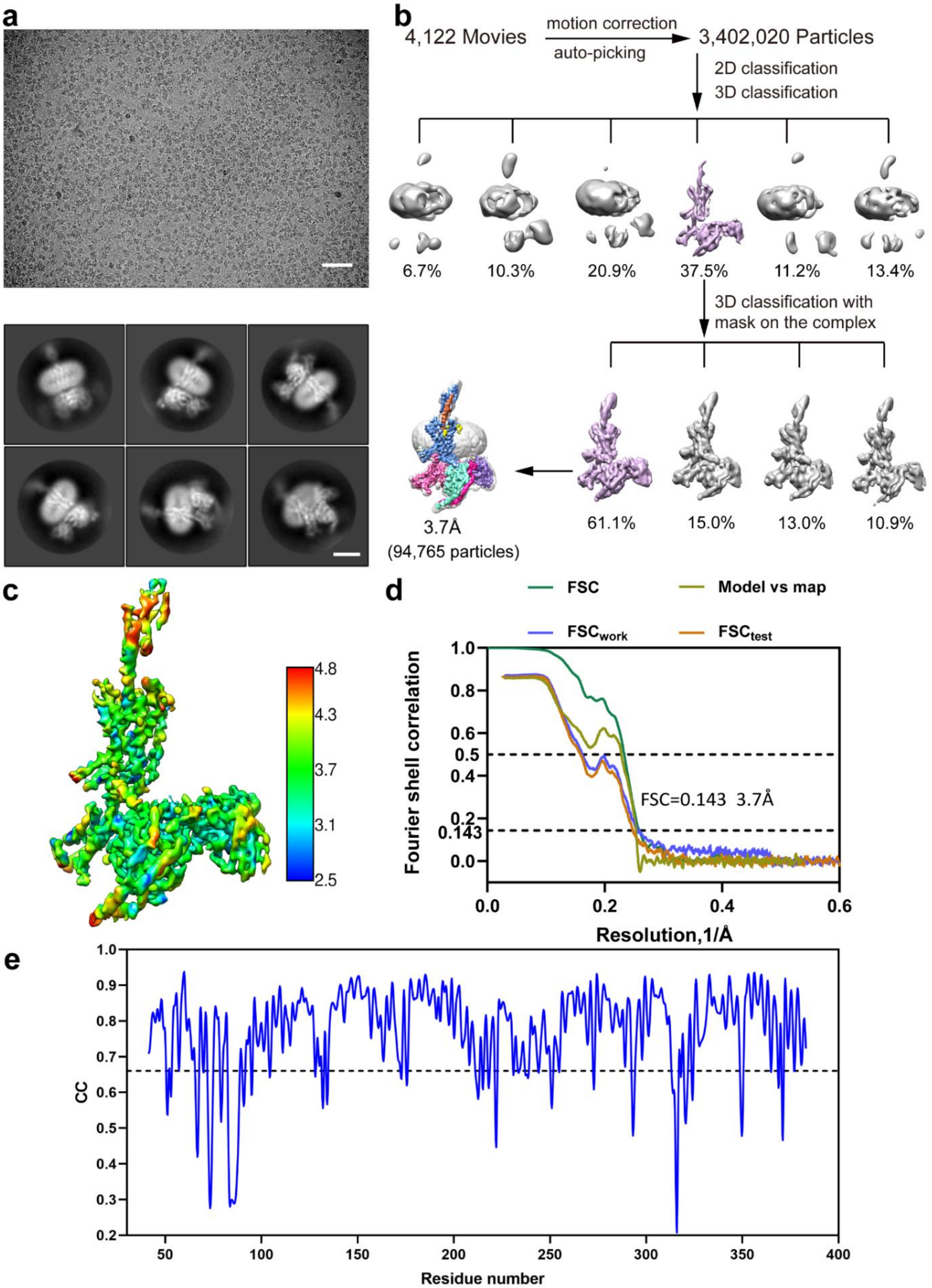

Supplementary Fig. 2. Flowchart of cryo-EM data analysis of the UCN1-CRF2R-G_{11} complex. (a) Top left, representative cryo-EM micrographs of the UCN1-CRF2R-G_{11} complex (scale bar: 50 nm); bottom left, representative 2D class averages showing distinct secondary structure features from different views (scale bar: 5 nm). **(b)** Flowchart of cryo-EM data analysis. **(c)** Cryo-EM map of the UCN1-CRF2R-G_{11} complex, colored by local resolution (Å) calculated using the Bsoft package. **(d)** "Gold-standard" FSC curve of the UCN1-CRF2R-G_{11} complex. **(e)** Per-residue CC plot of the CRF2R from the output of real-space refinement in Phenix 1.16.

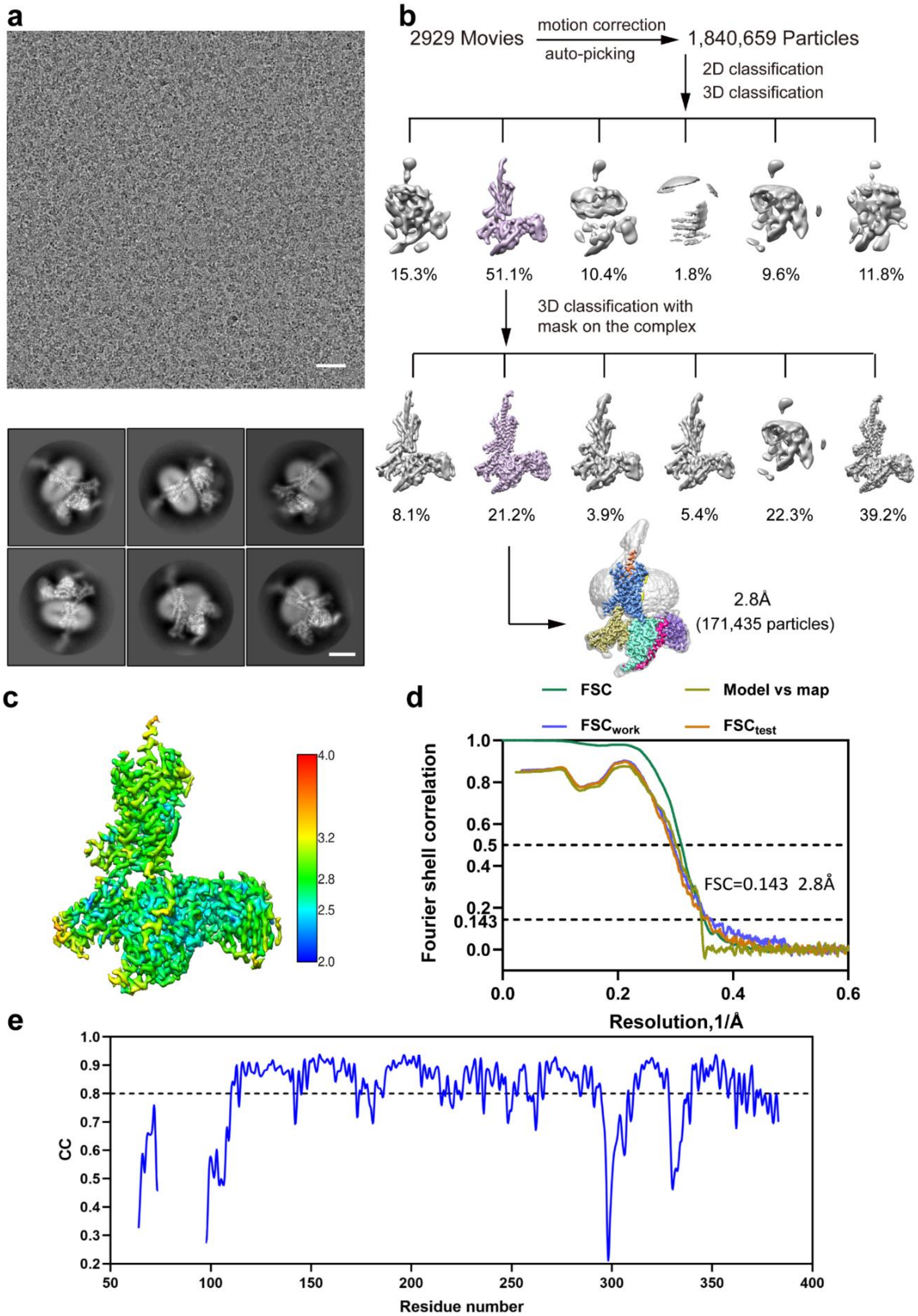

Supplementary Fig. 3. Flowchart of cryo-EM data analysis of the UCN1-CRF2R-G_o complex. (a) Top left, representative cryo-EM micrographs of the UCN1-CRF2R-G_o complex (scale bar: 50 nm) and bottom left, representative 2D class averages showing distinct secondary structure features from different views (scale bar: 5 nm). **(b)** Flowchart of cryo-EM data analysis. **(c)** Cryo-EM map of the UCN1-CRF2R-G_o complex, colored by local resolution (Å) calculated using the Bsoft package. **(d)** "Gold-standard" FSC curve of the UCN1-CRF2R-G_o complex. **(e)** Per-residue CC plot of the CRF2R from the output of real-space refinement in Phenix 1.16.

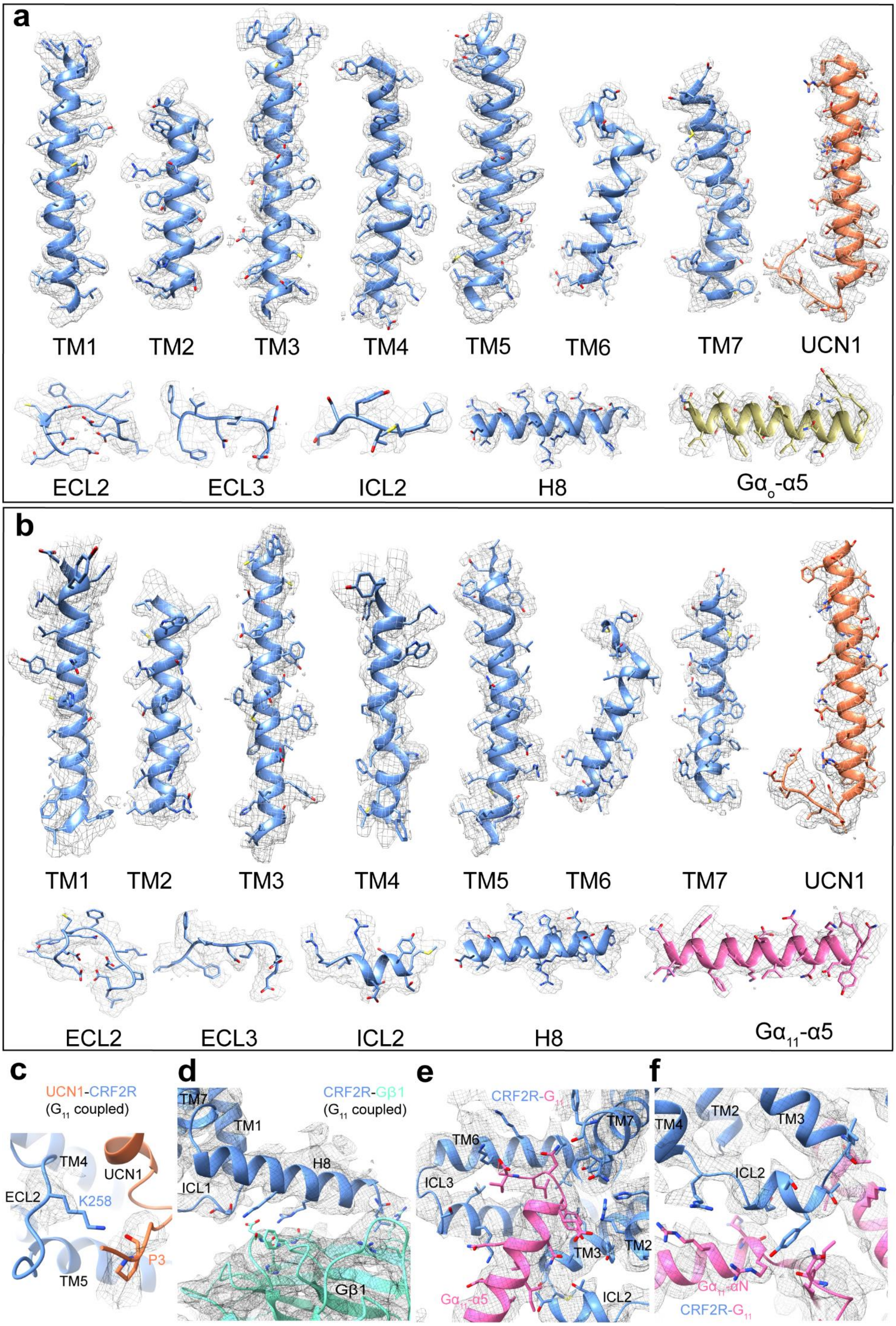

Supplementary Fig. 4 Cryo-EM density maps of the UCN1-CRF2R–G protein structures. Cryo-

EM density maps and the models of UCN1-CRF2R-G_o **(a)** and UCN1-CRF2R-G_{11} **(b)** complexes. All transmembrane helices, ECL2, ECL3, ICL2, H8, UCN1 and the α5 helix of the $G\alpha_o$ and $G\alpha_{11}$ are shown. **(c)** The density are shown as a surface mesh for $K258^{ECL2}$ (cornflower blue), which was modeled with Rosetta in the UCN1-CRF2R-G_{11} structure and forms an H-bond with $P3^{UCN1}$ (coral). **(d)** The density are shown as a surface mesh for the side chains of K372, and D379 of the receptor (cornflower blue), and R42 and D312 of Gβ1(aquamarine) in the UCN1-CRF2R-G_{11} complex, which were truncated in the structure and whose rotamers shown in this panel were from the Rosetta-refined model. **(e)** Receptor side chains and those of K345, D346, and E355 on Gα-α5 (hot pink) were truncated in the UCN1-CRF2R-G_{11} structure, whose rotamers shown in this panel were from the Rosetta-refined model and their density are shown as a surface mesh. **(f)** The side chains of receptor residues, and N198, I199, and K345 of G_{11} in this panel were truncated in the UCN1-CRF2R-G_{11} structure. Those residues shown here were prepared based on the Rosetta-refined model and the density are shown as a surface mesh to aid interpretation of the interactions between ICL2 and G_{11} (hot pink).

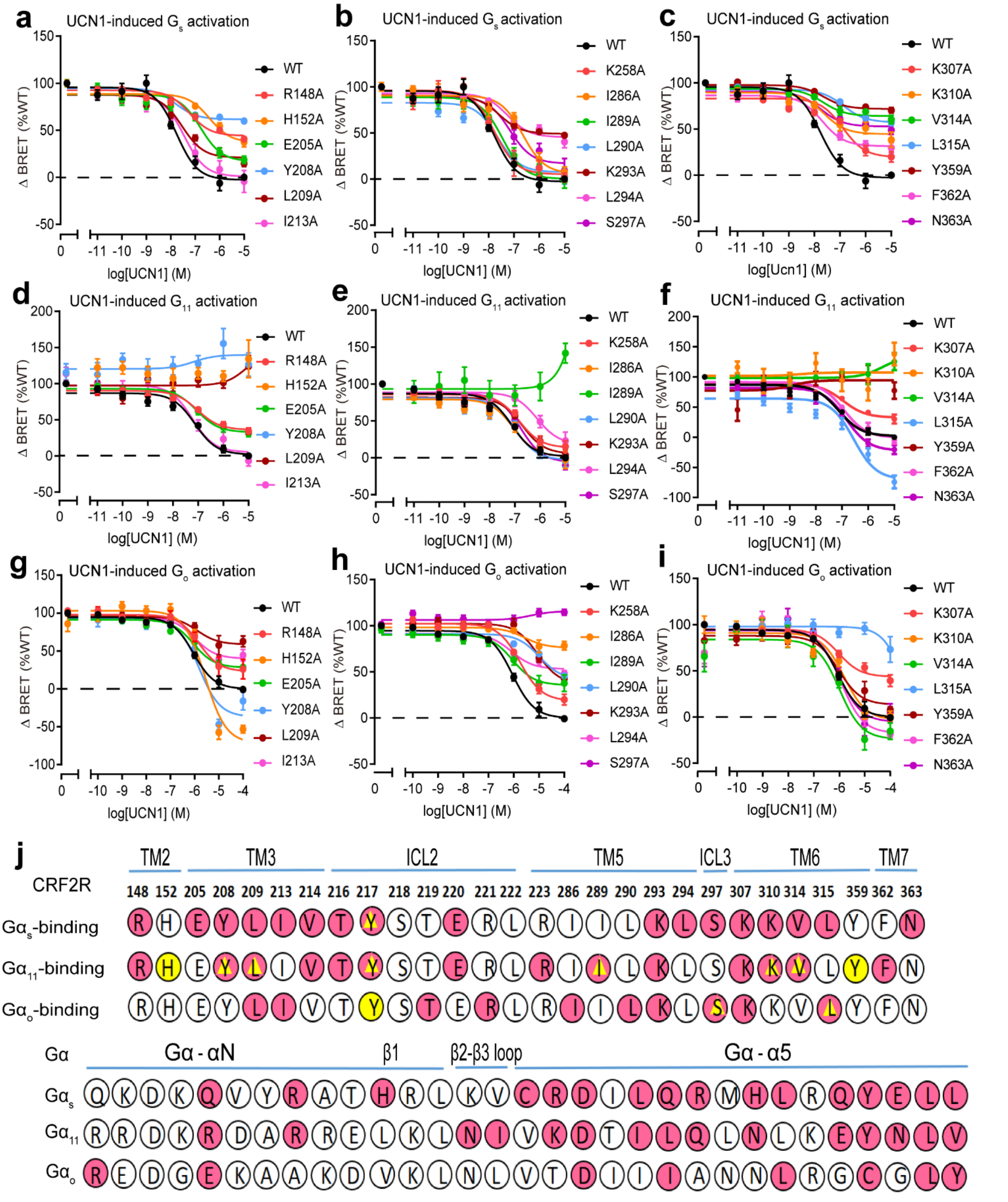

Supplementary Fig. 5 G protein activation and signaling assays of wild-type (WT) and mutant CRF2Rs and the schematic figure of important residues on CRF2R and G protein for formation of the G protein subtype-specific interactions. (a-i) G-protein activation of transmembrane helical mutants using a G protein dissociation assay**. (a-c)** the Gα$_s$-Gβγ dissociation assay **(d-f)** the Gα$_{11}$-Gβγ dissociation assay and **(g-i)** the Gα$_o$-Gβγ dissociation assay. Data from three independent experiments (n=3) are presented as mean ± SEM. **(j)** The important residues on CRF2R and G protein for formation of the G protein subtype-specific interactions are colored pink, and the yellow represents that alanine mutations completely abolished UCN1 potency on G protein signaling.

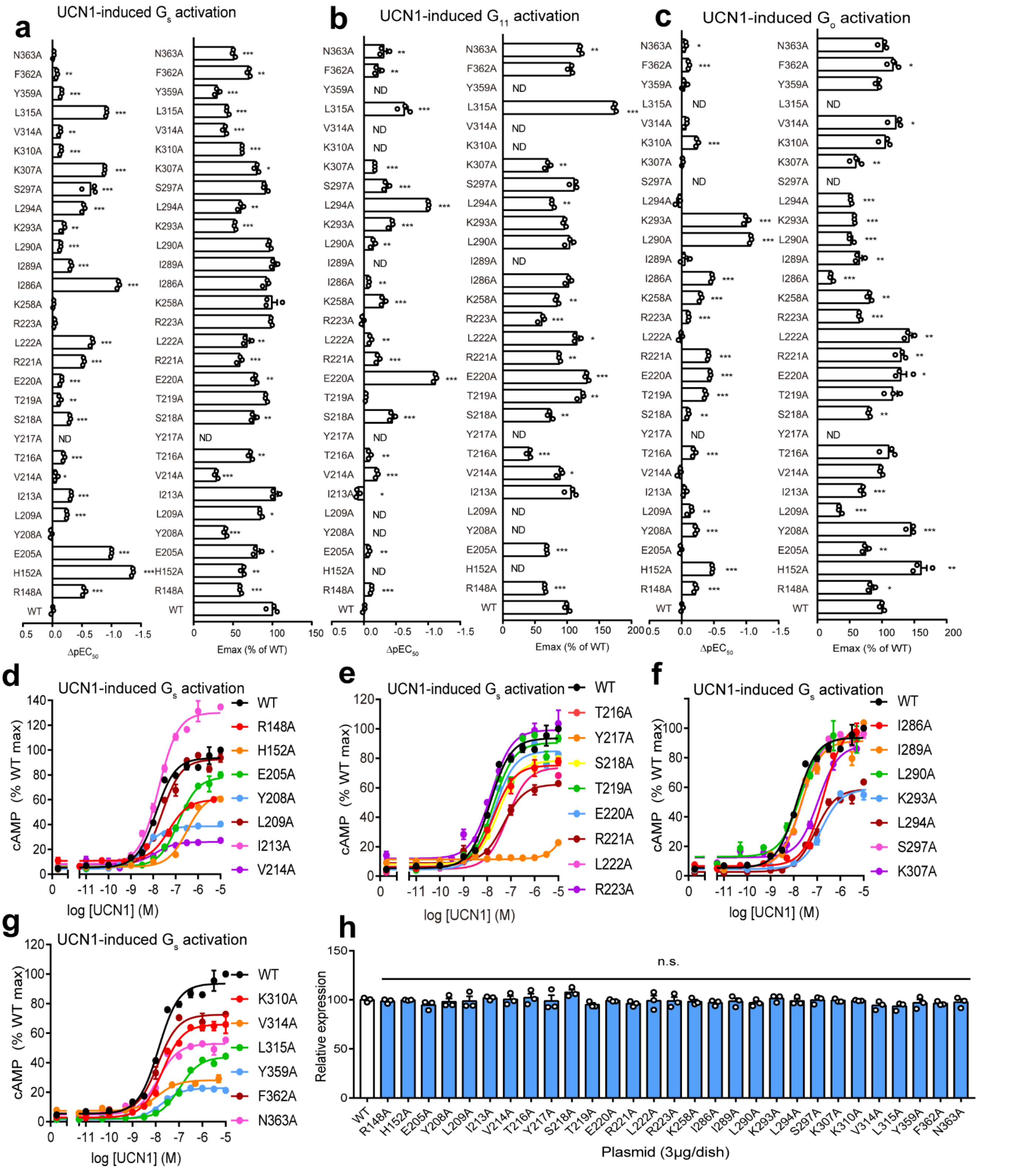

Supplementary Fig. 6 G protein activation and signaling assays of wild-type (WT) and mutant CRF2Rs and expression levels of CRF2R mutations in HEK293 cells. (a-c) Bars represent differences in calculated potency of UCN1(pEC50 [half maximal effective concentration]) for each mutation relative to WT of CRF2R (left) and the maximal response (right) of wild-type and mutants CRF2R on the Gα$_s$-Gβγ dissociation assay **(a)** Gα$_{11}$-Gβγ dissociation assay **(b)** and Gα$_o$-Gβγ dissociation assay **(c)** in HEK293 cells in response to UCN1 stimulation. The maximal responses of mutants CRF2R were normalized to that of WT CRF2R. Data from three independent experiments (n=3) are presented as mean ± SEM. Statistical differences between WT and mutations were determined by two-sided one-way ANOVA with Tukey's test. **P*<0.05; ***P*<0.01; ****P*<0.001. ND, not detectable. The exact *P* values are provided as a Source Data file. **(d-g)** UCN1-induced cAMP accumulation assay.

Data from three independent experiments (n=3) are presented as mean ± SEM. **(h)** Relative expression levels of CRF2R WT and alanine mutations were measured by cell surface ELISA assay. The expression levels of the mutations were normalized to that of the WT CRF2R. Data from three independent experiments (n=3) are presented as mean ± SEM. Statistical differences between WT and mutations were determined by two-sided one-way ANOVA with Tukey test. n.s., the mutant showed no significant difference compared to WT.

Supplementary Table 1. Cryo-EM data collection, refinement and validation statistics.

	UCN1–CRF2R–G_{11}–scFv16 complex (EMDB-26103) (PDB 7TRY)	UCN1–CRF2R–G_o–scFv16 complex (EMDB-26104) (PDB 7TS0)
**Data collection and processing**		
Magnification	46685	49310
Voltage (kV)	300	300
Electron exposure (e^-/Å^2)	80	64
Defocus range (μm)	-1.2 to -2.2	-1.5 to -2.3
Pixel size (Å)	1.045	1.014
Symmetry imposed	C1	C1
Initial particle images (no.)	3,402,020	1,840,659
Final particle images (no.)	94,765	171,435
Map resolution (Å)	3.7	2.8
FSC threshold	0.143	0.143
Map resolution range (Å)	2.5-5.5	2.0-4.0
**Refinement**		
Initial model used (PDB code)	6PB1/3N93	6PB1/3N93
Model resolution (Å)	3.7	2.8
FSC threshold	0.5	0.5
Model resolution range (Å)	5.0-7.8	2.2-5.0
Map sharpening B factor (Å^2)	-108.587	-74.9693
Model composition		
Non-hydrogen atoms	8400	9592
Protein residues	1221	1186
Lipids	0	9
B factors (Å^2)		
Protein	128.1	55.0
Lipids	N.A.	100.0
R.m.s. deviations		
Bond lengths (Å)	0.005	0.005
Bond angles (Å)	1.122	1.085
Validation		
MolProbity score	1.13	1.17
Clash score	3.34	3.85
Poor rotamers (%)	0.0	0.1
Ramachandran plot		
Favored (%)	98.31	98.54
Allowed (%)	1.69	1.46
Disallowed (%)	0.00	0.00

Supplementary Table 2. Effects of mutations on UCN1-induced activation of wild-type and mutant CRF2R.

Mutant	UCN1-induced G_s activation		UCN1-induced G_{11} activation		UCN1-induced G_o activation		Expression (% of WT)
	pEC_{50}±SEM	E_{max} (% of wt)	pEC_{50}±SEM	E_{max} (% of wt)	pEC_{50}±SEM	E_{max} (% of wt)	
WT	7.77±0.02	100.00±5.97	7.13±0.02	100.00±3.23	6.04±0.01	100.00±3.05	100.00±1.96
R148A	7.24±0.02	59.48±1.06	7.02±0.01	65.69±0.73	5.83±0.02	83.30±4.75	98.63±1.88
H152A	6.42±0.01	62.52±2.21	ND	ND	5.57±0.00	160.20±13.00	99.69±0.36
E205A	6.78±0.01	80.37±4.76	7.06±0.01	68.48±0.45	6.06±0.01	74.28±4.32	94.69±3.74
Y208A	7.80±0.01	40.44±1.65	ND	ND	5.82±0.01	144.78±5.41	98.04±4.79
L209A	7.53±0.01	85.10±1.58	ND	ND	5.91±0.04	34.83±2.39	98.74±6.77
I213A	7.46±0.01	103.48±4.28	7.23±0.03	106.04±7.59	5.99±0.03	69.61±2.31	101.77±1.68
V214A	7.71±0.02	28.74±1.81	6.93±0.02	88.92±4.16	6.08±0.03	98.70±1.92	100.42±4.81
T216A	7.58±0.01	72.12±2.22	7.05±0.02	40.79±2.93	5.85±0.02	110.33±10.36	102.17±5.93
Y217A	ND	ND	ND	ND	ND	ND	98.80±8.44
S218A	7.48±0.01	76.17±3.36	6.70±0.04	73.34±3.50	5.94±0.01	80.26±1.35	107.60±3.87
T219A	7.65±0.02	92.16±1.20	7.10±0.01	121.92±4.52	5.68±0.01	116.28±10.29	95.05±2.41
E220A	7.63±0.02	78.10±2.27	6.03±0.02	130.76±2.50	5.60±0.01	131.99±11.87	99.17±0.96
R221A	7.25±0.01	58.44±2.91	6.91±0.02	88.25±1.10	5.63±0.01	129.48±6.62	96.02±2.25
L222A	7.11±0.01	67.55±4.76	7.03±0.02	115.08±4.76	6.06±0.02	141.71±6.67	99.09±7.35
R223A	7.74±0.01	98.58±0.90	7.15±0.02	60.43±4.45	5.94±0.00	65.43±1.47	98.81±5.59
K258A	7.76±0.01	99.31±9.58	6.83±0.03	84.33±2.27	5.75±0.02	80.69±2.98	98.23±2.77
I286A	6.66±0.02	92.17±3.35	7.06±0.00	102.07±4.25	5.57±0.01	21.81±2.29	97.01±2.36
I289A	7.46±0.01	102.07±3.49	ND	ND	5.99±0.06	64.72±6.51	98.50±4.56
L290A	7.64±0.01	96.43±1.73	6.99±0.03	103.61±5.76	4.98±0.01	51.94±3.52	96.89±2.80
K293A	7.59±0.03	52.64±0.83	6.71±0.03	96.10±2.01	5.04±0.03	57.55±0.37	101.38±2.98
L294A	7.25±0.02	59.65±3.16	6.13±0.01	77.47±1.81	6.09±0.03	51.91±0.90	98.76±3.25
S297A	7.13±0.1	90.98±2.65	6.78±0.04	110.67±5.11	ND	ND	99.76±3.17
K307A	6.89±0.01	80.05±2.52	6.96±0.00	69.85±4.01	6.03±0.01	59.34±7.62	99.25±1.50
K310A	7.63±0.01	61.65±0.13	ND	ND	5.80±0.02	105.03±7.53	99.21±0.47
V314A	7.65±0.02	39.64±2.23	ND	ND	5.99±0.02	121.44±9.14	94.35±3.68
L315A	6.86±0.01	43.04±1.48	6.50±0.09	174.30±1.86	ND	ND	93.40±3.27
Y359A	7.62±0.01	29.69±2.90	ND	ND	6.00±0.04	93.79±2.91	96.95±4.98
F362A	7.70±0.01	70.14±1.74	6.92±0.05	105.46±3.36	5.93±0.01	117.04±7.32	96.30±1.73
N363A	7.76±0.01	50.85±1.61	6.82±0.07	120.69±1.90	5.99±0.02	101.46±5.94	97.57±4.38

Effects of mutations on UCN1-induced activation of wild-type and mutant CRF2R. pEC_{50} and E_{max} were calculated to determine the signaling efficiency and potency, respectively. The maximal response is reported as a percentage of the maximum effect at the WT. Data are from at least three independent experiments. ND, not detectable. Expression levels of CRF2R mutants. The relative expression levels of CRF2R in HEK293 cells were determined by ELISA assay using WT as the reference.

Supplementary Table 3. List of primer sequences for site-direct mutagenesis studies.

Oligonucleotides primer	Forward	Reverse
R148A	CTCCATCCGGTGTCTTGCGAATGTCATCCACTGGAATC	CAGTGGATGACATTCGCAAGACACCGGATGGAGCGCAGTG
H152A	CTTCGGAATGTCATCGCCTGGAATCTTATTACTACCTTTATAC	GTAATAAGATTCCAGGCGATGACATTCCGAAGACACCGGATG
E205A	TTTTGGATGTTTGTTGCAGGATGCTATCTGCACACCGCCATC	TGTGCAGATAGCATCCTGCAACAAACATCCAAAAGAAATTAG
Y208A	GTTTGTTGAAGGATGCGCTCTGCACACCGCCATCGTCATGAC	GACGATGGCGGTGTGCAGAGCGCATCCTTCAACAAACATCCAAAAG
L209A	TGTTGAAGGATGCTATGCGCACACCGCCATCGTCATGAC	GACGATGGCGGTGTGCGCATAGCATCCTTCAACAAACATC
I213A	GCTATCTGCACACCGCCGCCGTCATGACATATTCTACCGAAAGAC	GTAGAATATGTCATGACGGCGGCGGTGTGCAGATAGCATCCTTCAAC
V214A	TCTGCACACCGCCATCGCCATGACATATTCTACCGAAAGACTGCGC	CGGTAGAATATGTCATGGCGATGGCGGTGTGCAGATAGCATCCTTC
T216A	ACACCGCCATCGTCATGGCATATTCTACCGAAAGACTGCGC	TCTTTCGGTAGAATATGCCATGACGATGGCGGTGTGCAGATAGC
Y217A	CACCGCCATCGTCATGACAGCTTCTACCGAAAGACTGCGCAAGTG	GCAGTCTTTCGGTAGAAGCTGTCATGACGATGGCGGTGTGCAG
S218A	CATCGTCATGACATATGCTACCGAAAGACTGCGCAAGTGCCTG	TGCGCAGTCTTTCGGTAGCATATGTCATGACGATGGCGGTGTG
T219A	GTCATGACATATTCTGCCGAAAGACTGCGCAAGTGCCTGTTTTTG	CTTGCGCAGTCTTTCGGCAGAATATGTCATGACGATGGCG
E220A	ATGACATATTCTACCGCAAGACTGCGCAAGTGCCTGTTTTTG	CACTTGCGCAGTCTTGCGGTAGAATATGTCATGACGATGGCG
R221A	ACATATTCTACCGAAGCACTGCGCAAGTGCCTGTTTTTGTTC	CAGGCACTTGCGCAGTGCTTCGGTAGAATATGTCATGACGATG
L222A	ATATTCTACCGAAAGAGCGCGCAAGTGCCTGTTTTTGTTCATC	AAAACAGGCACTTGCGCGCTCTTTCGGTAGAATATGTCATGAC
R223A	ATATTCTACCGAAAGACTGGCCAAGTGCCTGTTTTTGTTCATCGGCTG	GAACAAAAACAGGCACTTGGCCAGTCTTTCGGTAGAATATGTCATGACG
K258A	AGTGCTGGTTCGGGGCAGAGCCCGGCGACCTTGTCGACTAC	AAGGTCGCCGGGCTCTGCCCCGAACCAGCACTGCTCGTTCTC
I286A	GTGTTCCTCTTCAACGCTGTGCGCATTCTGATGACAAAGCTG	CATCAGAATGCGCACAGCGTTGAAGAGGAACACGAAGTTTATAAG
I289A	TTCAACATTGTGCGCGCTCTGATGACAAAGCTGCGAGCCAGC	AGCTTTGTCATCAGAGCGCGCACAATGTTGAAGAGGAACACGAAG
L290A	AACATTGTGCGCATTGCGATGACAAAGCTGCGAGCCAGCAC	TCGCAGCTTTGTCATCGCAATGCGCACAATGTTGAAGAGGAAC
K293A	GCGCATTCTGATGACAGCGCTGCGAGCCAGCACAACATCAGAG	TGTGCTGGCTCGCAGCGCTGTCATCAGAATGCGCACAATGTTG
L294A	ATTCTGATGACAAAGGCGCGAGCCAGCACAACATCAGAGACTATAC	GTTGTGCTGGCTCGCGCCTTTGTCATCAGAATGCGCACAAT
S297A	ACAAAGCTGCGAGCCGCCACAACATCAGAGACTATACAGTACCG	TAGTCTCTGATGTTGTGGCGGCTCGCAGCTTTGTCATCAGAATGC
K307A	GACTATACAGTACCGGGCGGCAGTTAAGGCCACTCTGGTCCTTTTG	GAGTGGCCTTAACTGCCGCCCGGTACTGTATAGTCTCTGATGTTG
K310A	GTACCGGAAGGCAGTTGCGGCCACTCTGGTCCTTTTGCCACTGC	AAGGACCAGAGTGGCCGCAACTGCCTTCCGGTACTGTATAGTCTC
V314A	AGTTAAGGCCACTCTGGCCCTTTTGCCACTGCTGGGGATCAC	AGCAGTGGCAAAAGGGGCCAGAGTGGCCTTAACTGCCTTCCG
L315A	TAAGGCCACTCTGGTCGCTTTGCCACTGCTGGGGATCACTTAC	CAGCAGTGGCAAAGCGACCAGAGTGGCCTTAACTGCCTTCCG
Y359A	TTTTGTATCAGTGTTCGCTTGCTTCTTTAATGGGGAGGTGCGGTC	CTCCCCATTAAAGAAGCAAGCGAACACTGATACAAAAAAGCCTTG

F362A	TGTTCTATTGCTTCGCTAATGGGGAGGTGCGGTCTGCAGTGCG	CGCACCTCCCCATTAGCGAAGCAATAGAACACTGATACAAAAAAGC
N363A	GTTCTATTGCTTCTTTGCTGGGGAGGTGCGGTCTGCAGTGCG	GACCGCACCTCCCCAGCAAAGAAGCAATAGAACACTGATAC
