# Supplementary material for: Structure insights into selective coupling of G protein subtypes by a class B G protein-coupled receptor
Source: Nat Commun. 2022 Nov 5;13:6670. doi: 10.1038/s41467-022-33851-3 (PMC9637140; doi:10.1038/s41467-022-33851-3)
Supplement: Supplementary file 3 — Description of Additional Supplementary Files [file 41467_2022_33851_MOESM3_ESM.pdf]

Supplementary Data 1

The model of the CRF2R-G_{11} complex refined with Rosetta refinement techniques against the cryo-EM map, with improved the atomic details, chemically optimized side chain rotamers and the global protein geometry.
