# Supplementary material for: Structure insights into selective coupling of G protein subtypes by a class B G protein-coupled receptor
Source: Nat Commun. 2022 Nov 5;13:6670. doi: 10.1038/s41467-022-33851-3 (PMC9637140; doi:10.1038/s41467-022-33851-3)
Supplement: Supplementary file 5 — Reporting Summary [file 41467_2022_33851_MOESM5_ESM.pdf]

nature portfolio

Corresponding author(s): Li-hua Zhao
H. Eric Xu

Last updated by author(s): Sep 22, 2022

Reporting Summary

Nature Portfolio wishes to improve the reproducibility of the work that we publish. This form provides structure for consistency and transparency in reporting. For further information on Nature Portfolio policies, see our Editorial Policies and the Editorial Policy Checklist.

Statistics

For all statistical analyses, confirm that the following items are present in the figure legend, table legend, main text, or Methods section.

n/a	Confirmed	
☐	☒	The exact sample size (*n*) for each experimental group/condition, given as a discrete number and unit of measurement
☐	☒	A statement on whether measurements were taken from distinct samples or whether the same sample was measured repeatedly
☐	☒	The statistical test(s) used AND whether they are one- or two-sided<br>*Only common tests should be described solely by name; describe more complex techniques in the Methods section.*
☒	☐	A description of all covariates tested
☒	☐	A description of any assumptions or corrections, such as tests of normality and adjustment for multiple comparisons
☐	☒	A full description of the statistical parameters including central tendency (e.g. means) or other basic estimates (e.g. regression coefficient) AND variation (e.g. standard deviation) or associated estimates of uncertainty (e.g. confidence intervals)
☐	☒	For null hypothesis testing, the test statistic (e.g. *F*, *t*, *r*) with confidence intervals, effect sizes, degrees of freedom and *P* value noted<br>*Give P values as exact values whenever suitable.*
☒	☐	For Bayesian analysis, information on the choice of priors and Markov chain Monte Carlo settings
☒	☐	For hierarchical and complex designs, identification of the appropriate level for tests and full reporting of outcomes
☒	☐	Estimates of effect sizes (e.g. Cohen's *d*, Pearson's *r*), indicating how they were calculated

Our web collection on statistics for biologists contains articles on many of the points above.

Software and code

Policy information about availability of computer code

Data collection	Automated data collection on the Titan Krios equipped with a Gatan K3 was performed using serialEM 3.7 (UCN1–CRF2R–G11 complex).<br>Automated data collection on the Titan Krios equipped with a Gatan K2 was performed using serialEM 3.8 (UCN1–CRF2R–Go complex).
Data analysis	The following software was used in this study:PyMol 2.4.0, MotionCor2.1, GGctfv1.06, Relion3.0., UCSF Chimera v1.1, Phnix 1.16.Coot 0.9 EL, GraphPad Prism 8.0, Bsoft package(v.2.0.7).

For manuscripts utilizing custom algorithms or software that are central to the research but not yet described in published literature, software must be made available to editors and reviewers. We strongly encourage code deposition in a community repository (e.g. GitHub). See the Nature Portfolio guidelines for submitting code & software for further information.

Data

Policy information about availability of data

All manuscripts must include a data availability statement. This statement should provide the following information, where applicable:

- Accession codes, unique identifiers, or web links for publicly available datasets
- A description of any restrictions on data availability
- For clinical datasets or third party data, please ensure that the statement adheres to our policy

All relevant data are available from the corresponding authors upon reasonable request. The raw data underlying Figs. 4f, 4g, 4h and supplementary Figs. 1a–b, 5a–i, 6a–h are provided as a Source Data file. Cryo-EM maps generated in this study have been deposited in the Electron Microscopy Data Bank under accession codes:

EMD-26103 (G11-bound CRF2R receptor), EMD-26104 (Go-bound CRF2R receptor). The atomic coordinates generated in this study have been deposited in the Protein Data Bank under accession codes: 7TRY (G11-bound CRF2R receptor) and 7TS0 (Go-bound CRF2R receptor). Due to the limitation of the map resolution, many side chains of the G11-bound CRF2R structure were truncated before PDB deposit. The whole structural model with all Rosetta optimized side chains is included as a supplementary data file.

Human research participants

Policy information about studies involving human research participants and Sex and Gender in Research.

Reporting on sex and gender	N/A
Population characteristics	N/A
Recruitment	N/A
Ethics oversight	N/A

Note that full information on the approval of the study protocol must also be provided in the manuscript.

Field-specific reporting

Please select the one below that is the best fit for your research. If you are not sure, read the appropriate sections before making your selection.

☒ Life sciences ☐ Behavioural & social sciences ☐ Ecological, evolutionary & environmental sciences

For a reference copy of the document with all sections, see nature.com/documents/nr-reporting-summary-flat.pdf

Life sciences study design

All studies must disclose on these points even when the disclosure is negative.

Sample size	For cryo-EM data, images were collected until the resolution and 3D reconstruction converges. For all the functional assay, no statistical approaches were used to predetermine the sample size. All functional data were obtained from at least three independent experiments to ensure each data point was repeatable.
Data exclusions	No data were excluded.
Replication	For all functional assays, each experiment was repeated independently at least three times and all attempts at replication were successful.
Randomization	Randomization is not relevant to this study,as protein samples are not required to be divided into experimental groups in the structural studies, and no animals or human research participants are involved in this study.
Blinding	Blinding is not relevant to this study, since all the data were collected automatically.

Reporting for specific materials, systems and methods

We require information from authors about some types of materials, experimental systems and methods used in many studies. Here, indicate whether each material, system or method listed is relevant to your study. If you are not sure if a list item applies to your research, read the appropriate section before selecting a response.

Materials & experimental systems

n/a	Involved in the study	
☐	☒	Antibodies
☐	☒	Eukaryotic cell lines
☒	☐	Palaeontology and archaeology
☒	☐	Animals and other organisms
☒	☐	Clinical data
☒	☐	Dual use research of concern

Methods

n/a	Involved in the study	
☒	☐	ChIP-seq
☒	☐	Flow cytometry
☒	☐	MRI-based neuroimaging

March 2021

Antibodies

Antibodies used	Antibody used: Anti-Flag (Sigma-Aldrich, Cat# F1804). Secondary anti-mouse antibody (Thermo Fisher, Cat #A4416). The primary antibody was used in 1:1000 dilution, and the secondary antibody was used in 1:5000 dilution.
Validation	All antibodies used are commercially purchased and have been validated by the vendors. All antibodies are well characterized and were applied according to data sheet information details.<br>Monoclonal anti-Flag antibody: https://www.sigmaaldrich.com/catalog/product/sigma/f1804;<br>secondary anti-mouse antibody: https://www.thermofisher.com/cn/zh/antibody/product/Goat-anti-Mouse-IgG-H-L-CrossAdsorbed-Secondary-Antibody-Polyclonal/A-21235;

Eukaryotic cell lines

Policy information about cell lines and Sex and Gender in Research

Cell line source(s)	Sf9 (Invitrogen)<br>HEK293 cells were obtained from Cell Resource Center of Shanghai Institute for Biological Sciences (Chinese Academy of Sciences, Shanghai, China)
Authentication	No authentication required.
Mycoplasma contamination	Cell lines were tested and free from mycoplasma contamination.
Commonly misidentified lines<br>(See ICLAC register)	No commonly misidentified cell lines were used.
